# Cortical Entropy, Mutual Information and Scale-Free Dynamics in Waking Mice

**DOI:** 10.1093/cercor/bhw200

**Published:** 2016-09-19

**Authors:** Erik D. Fagerholm, Gregory Scott, Woodrow L. Shew, Chenchen Song, Robert Leech, Thomas Knöpfel, David J. Sharp

**Affiliations:** 1 The Computational, Cognitive and Clinical Neuroimaging Laboratory, The Centre for Neuroscience, The Division of Brain Sciences, Imperial College London, Hammersmith Hospital Campus, Du Cane Road, London, W12 0NN, UK; 2 University of Arkansas, Department of Physics, Fayetteville, AR 72701, USA; 3 Division of Brain Sciences, Department of Medicine, Imperial College London, Hammersmith Hospital Campus, Du Cane Road, London, W12 0NN, UK; 4 Centre for Neurotechnology, Institute of Biomedical Engineering, Imperial College London, South Kensington, London SW7 2AZ, UK

**Keywords:** scale-free dynamics, information capacity, information transmission, anesthesia, voltage imaging

## Abstract

Some neural circuits operate with simple dynamics characterized by one or a few well-defined spatiotemporal scales (e.g. central pattern generators). In contrast, cortical neuronal networks often exhibit richer activity patterns in which all spatiotemporal scales are represented. Such “scale-free” cortical dynamics manifest as cascades of activity with cascade sizes that are distributed according to a power-law. Theory and *in vitro* experiments suggest that information transmission among cortical circuits is optimized by scale-free dynamics. *In vivo* tests of this hypothesis have been limited by experimental techniques with insufficient spatial coverage and resolution, i.e., restricted access to a wide range of scales. We overcame these limitations by using genetically encoded voltage imaging to track neural activity in layer 2/3 pyramidal cells across the cortex in mice. As mice recovered from anesthesia, we observed three changes: (a) cortical information capacity increased, (b) information transmission among cortical regions increased and (c) neural activity became scale-free. Our results demonstrate that both information capacity and information transmission are maximized in the awake state in cortical regions with scale-free network dynamics.

## Introduction

The exchange of information among distant regions of cortex is essential to cortical function. What determines the efficacy of information flow between a pair of connected cortical regions? A fundamental constraint on such information exchange is imposed by the dynamical regime in which each of the two regions operates. Certain dynamical regimes allow a neural circuit to generate a large and varied repertoire of activity patterns, while other dynamical regimes result in a smaller activity repertoire (Shew et al. 2011). A high degree of information exchange between two cortical regions requires that both have large repertoires of activity patterns to encode the exchanged information. What kind of dynamical regime facilitates such large activity repertoires and information exchange?

Here we adopt information theory as a framework for studying how much information is represented and transmitted by neural circuits (Shannon 1948; Rieke 1997). In this framework, the repertoire of activity patterns that a cortical network can produce is quantified by its entropy (H) (Shew et al. 2011), sometimes referred to as “information capacity.” The transmission of information between pairs of cortical regions can be quantified by mutual information (MI).

We investigate a wide range of cortical dynamical regimes as mice recover from anesthesia. Previous studies have found that loss of consciousness is associated with a collapse in the repertoire of cortical activity patterns (H), as well as a breakdown in cortical interactions (MI) (Alkire et al. 2008). In our previous work, we observed a shift from predominantly large-scale activity patterns to scale-free patterns of activity during recovery from anesthesia (Scott et al. 2014). Previous *in vitro* studies and computational model work suggest that the diversity and complexity of scale-free dynamics may be optimal for achieving high information capacity (entropy) and transmission (mutual information) (Shew et al. 2011). However, this hypothesis has not been tested *in vivo*, nor at scales large enough to determine the relevance to inter-regional cortical information flow. Here we address this question using genetically encoded voltage indicator (GEVI) optical imaging, to directly measure activity pattern repertoires and information exchange across a large part of mouse cortex with high resolution. We examine changes in the activity repertoire and information transmission during changes in the cortical dynamical regime as the mouse recovers from anesthesia.

## Materials and Methods

### Animals

Two groups of mice were used.

Group 1 consisted of three wild type mice, which were electroporated three times *in utero* (E14.5–E15.5) with the pCAG-voltage-sensitive fluorescent protein (VSFP) Butterfly 1.2 plasmid (Akemann et al. 2012, 2013), resulting in the expression of the Butterfly 1.2 VSFP in layer 2/3 pyramidal cells in one hemisphere. Experimental procedures for Group 1 were approved by the Institutional Animal Care and Use Committee of the RIKEN Wako Research Centre (Japan) and were conducted according to the US National Institutes of Health guidelines for animal research.

Group 2 consisted of two triple transgenic (Ai78(TITL-VSFPB)-D; Camk2a-tTA; Rasgrf2-2A-dCre) mice that selectively expressed the Butterfly 1.2 VSFP in pyramidal neurons of cortical layer 2/3 in both hemispheres (Madisen et al. 2015).

All mice in Groups 1 and 2 (aged 2–6 months, either sex) were under surgical anesthesia for the entire cranial window implantation surgery as described previously (Akemann et al. 2012, 2013). In brief, a head post was implanted onto the thinned mouse skull and secured using a self-cure adhesive resin cement (Super-Bond C&B, Sun Medical, Japan). The thinned skull was reinforced by a cover glass using a cyanoacrylate adhesive (group 1) (Drew et al. 2010) or a layer of Super-Bond C&B topped by a thin layer of clear nail polish (group 2) (Sofroniew et al. 2015). The mice underwent voltage imaging after at least 48 hours recovery from surgery, being head-fixed via implanted head post in a custom-made stereotaxic frame, with body temperature controlled and maintained at 37 °C by means of a feedback-controlled heat pad (Fine Science Tools). Experimental procedures for Group 2 were performed in accordance with the UK Animal Scientific Procedures Act (1986) at Imperial College London under Home Office Personal and Project licenses following appropriate ethical review.

Animals were experienced in recovering from anesthesia under the scope. At some point during this recovery, the animals went from a resting awake state to an active state, in which they have a drive to explore and walk. In the active state movement artifacts can occur, but these are easily recognized as positively correlated changes in the fluorescence recorded by the two cameras, as opposed to the negatively correlated optical signals that represent membrane voltage transients. However, in the present study only data obtained in the anesthetized and resting awake states were included in the analysis, minimizing the chance of movement artifacts.

### Voltage Imaging

Group 1 was imaged after being re-anesthetized with pentobarbital sodium (40 mg/kg i.p.). Group 2 was imaged in a fully awake state, at least 48 hours after sedation. Image acquisition for both groups of mice was performed with a dual emission wide-field epifluorescence microscope equipped with two synchronized CCD cameras (Sensicam, PCO), using high-power halogen lamps (Moritex, BrainVision) and optics (Semrock). The voltage imaging signal was calculated as the ratio of mKate2 to mCitrine fluorescence, taken after offset subtraction and equalization of heartbeat-related modulation of fluorescence. Image sequences of 60s duration followed by 60s pauses were acquired at 50 Hz, with 320 × 240 pixel resolution (Akemann et al. 2012).

### Data Preprocessing

All data were baseline normalized on a pixel-wise level, i.e., each pixel's baseline is the average over its values, for each 60s image sequence. Each 60s dataset was temporally smoothed using a sliding window to average pixel activity across 4 consecutive time points and then spatially smoothed using an 8 × 8 pixel averaging filter. Data were then high-pass filtered at 0.5 Hz in order to reduce the effect of slow trends in the baseline signal that may cause artificial (i.e., non-neural) correlations (Akemann et al. 2012). The first 10s of each image sequence were discarded to remove possible contribution from environmental cues present at the start of each imaging sequence (e.g., shutter noise and excitation light). Subsequent analyses were constrained to pixels within masks, drawn by hand for each mouse, which defined the extents of the bone window. We refined these masks by excluding regions with poor signal-to-noise ratios, defined as those pixels in which the protein expression (estimated as time-averaged absolute fluorescence intensity) was less than 50% of the maximum level across the field of view for each mouse. Imaging data were analyzed with Matlab using the Image and Signal Processing Toolboxes (Mathworks) and ImagePro 6.2 image processing software.

### Noise Datasets

We generated noise on a pixel-wise level with the same power spectrum as the ratio image data. These noise datasets were then passed through the same preprocessing pipeline as the experimental data. By showing null results for these noise data, we eliminate the possibility that the preprocessing pipeline and/or changes in the power spectrum are responsible for the relationships observed.

### Cascade Detection and Statistics

Cascades were detected as spatiotemporally contiguous clusters of active pixels (Tagliazucchi et al. 2012; Scott et al. 2014). Cascade detection was performed both across the entire image and also for regional subdivisions of the image. A pixel was defined as “active” at times when the voltage signal crossed above a threshold of +1 S.D. from below. A positive threshold was chosen as positive deflections of our optical signals indicate population depolarization (Akemann et al. 2012). Cascade detection results were previously tested for robustness between +0.5 and +1.5 S.D. (Scott et al. 2014). The cumulative event count at +1 S.D. for the entire recording area across all mice was 240 ± 12 per mouse per second.

Clusters of active pixels were identified based on detection of connected pixels in a coactive first neighbors graph. Cascades were then defined as starting with the activation of a previously inactive cluster and continuing while at least one contiguous cluster was active in the next time point. We defined the size of a cascade (z) as the number of active pixels comprising the cascade. The shape of the cascade size distribution changed systematically as animals awoke from anesthesia. In the awake resting state, the distribution was close to a power-law with exponent −1.5. To parameterize these changes, cascade size probability distributions were compared to a power law with exponent −1.5 using a measure κ (Shew et al. 2009; Yang et al. 2012). Thus, we do not interpret κ as a statistical test confirming a power-law distribution. Rather, κ is a measure of deviation from a power-law. In brief, to compute κ for one dataset, one first obtains a cumulative probability distribution function (CDF) of cascade sizes. Second, the distance between the observed CDF and a reference CDF is calculated at 10 equally spaced points, where the reference is a perfect power law with exponent −1.5 and κ is defined as 1 plus the average of the 10 differences. Our choice to use a reference power-law with an exponent of −1.5 in the calculation of κ was based both on theory (Larremore et al. 2012) as well as our previous work (Scott et al. 2014). However, one potential limitation of the κ metric is that cascade sizes could be distributed according to a power-law with an exponent other than −1.5, which would result in κ deviating from unity.

### k-means Clustering

In order to assess the repertoire of cortical brain states we applied a k-means clustering algorithm to the point-process data from the entire imaged area to produce a time course of cortical states. Prior to clustering, image sequences were spatially downsampled by a factor of 2 using interpolation with a box-shaped kernel, in order to reduce computational demands. We performed clustering separately on each 50s image sequence in order to eliminate bias in the clustering algorithm due to varying proportions of data from different brain states. The analyses were repeated with k = 10,50,200 clusters. For each resulting state time course, we quantified the repertoire of states by calculating the state visitation entropy (H_state_) of the probability distribution p_i_ as follows:

1 $$H_{state}=-\overset{k}{\underset{i=1}{\sum }}p_{i}log_{2}p_{i}$$

where *p*_*i*_ is the probability of the system being observed in state *i*, for *i* = 1,2,…,*k*. The probabilities used to calculate state visitation entropy are calculated separately for each 50s window. Using the state time courses, a first-order Markov model was used to create a state transition probability distribution of moving from state *i* to state *j*, where *i,j* = 1,2,…,*k*. The state transition entropy H_trans_ was then calculated using [1].

### Regional Entropy and Mutual Information

The point-process image sequences were divided into 8 × 8 pixel regions. An event was defined at each time point for each region with 1 bit per pixel. A bit was set to 1 if the corresponding pixel was active during the event and 0 otherwise. The entropy of this set of patterns was calculated for each region using [1].

The information transmission for a given region was defined as the sum of its mutual information (MI) with all other regions (Cover and Thomas 2012). The presence of mutual information between disparate regions of the cortex should be regarded as a somewhat generalized form of “correlation”, indicating encoding overlapping information content without shedding light on the neuronal code or the mechanism of information transport. MI is by definition a nonnegative quantity. As such, for finite size samples, even independent random variables will have positive MI, rendering interpretation difficult. To account for this, we adopt an approach similar to that used for “adjusted mutual information” and subtract from our MI measurements a control value of MI (Vinh et al. 2010). We calculate the control MI as above, but with a randomized order of states for one of the variables (Margolin et al. 2006). The control MI has values near zero (and can take negative values) for insignificant levels of MI and is positive for significant levels of MI. We exclude values of MI calculated between pairs of regions that are closer to one another than 10% of the maximum extent of the imaged cortex. This is to reduce the possibility of spurious correlations arising due to spatial smoothing operations in the preprocessing pipeline.

Regional information capacity and transmission were also calculated for different spatial extents, spatial resolutions and time steps: (a) 5 × 5 square regions, (b) 8 × 8 square regions, (c) half spatial resolution data and (d) half temporal resolution data. We use these different spatiotemporal definitions in order to demonstrate that the results are not dependent on a particular combination of analysis parameters.

## Results

### Voltage Imaging and Cascade Detection

**Figure 1.**
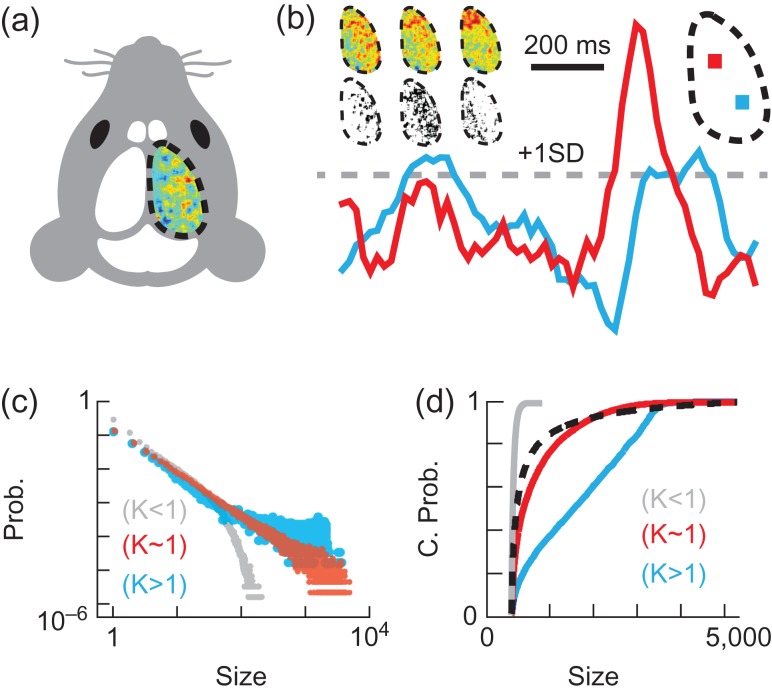
Experimental setup and data analysis. (*a*) Voltage map for 20 ms over one cortical hemisphere in a head-fixed mouse with trans-cranial window. (*b*) Voltage traces from two pixels (each covering a 33 × 33 μm cortical area) in the regions with corresponding colors (top right). 20 ms of averaged activity from normalized data are shown at three time points, together with their corresponding point-process images (top left). (*c*) Example cascade size (pixels) probability distributions with few large cascades (κ < 1, grey, noise), near power law distribution (κ ≈ 1, red, awake), and abundant large cascades (κ > 1, blue, anesthetized). (*d*) Cumulative probability distributions for the same examples as in (c), together with a reference distribution with exponent −1.5 (black dotted line), the proximity to which is quantified by κ.

In order to gain insight into information transmission among multiscale cortical circuits, we performed trans-cranial voltage imaging using a voltage-sensitive fluorescent protein (VSFP) in intact head-fixed mice (Fig. 1*a*) (Akemann et al. 2010, 2012). This wide-field epifluorescence imaging approach captures the membrane voltage averaged over tissue volumes that are projected onto each pixel, allowing neural activity to be recorded across the cortex with high spatiotemporal resolution (<100 μm, spatially oversampled at 33 × 33 μm^2^; limited by light scattering, 20 ms). We imaged mice recovering from pentobarbital anesthesia (40 mg/kg i.p., labels A1, A2, A3), as well as fully awake mice, imaged at least 48 hours after sedation (labels R1, R2) (Materials and Methods).

Our data analysis begins with the creation of a point-process from the voltage imaging data. We labeled all pixels in sequences of normalized voltage maps (240 × 320 pixels, 2500 frames per sequence, recorded at 50 Hz) as active when the voltage signal crossed a threshold of +1 S.D. from below, thus creating a point-process (Fig. 1*b*). This point-process transformation results in a given pixel being labeled as active only at the time point in which it first crosses the threshold. As such, a region that shows high activity in the normalized voltage map snapshot will not necessarily correspond to activity in the point-process image at the corresponding time point.

We used this point-process data in two different ways. First, we assessed changes in the cortical dynamics in the context of scale-free dynamics. Second, we used the point-process data to assess changes in information processing within and among different cortical regions. By combining these two types of data analysis, we ultimately identify which cortical states are optimized for information processing.

To assess changes in cortical state, we analyzed cascades of active pixels in the point-process data, similar to previous studies (Tagliazucchi et al. 2012; Scott et al. 2014). A cascade was defined as spatiotemporally contiguous clusters of active pixels, i.e., two active pixels that are immediate neighbors in space and time are defined as belonging to the same cascade. For comparison, we also explored an alternative definition of cascades based only on temporal contiguity of active pixels as in traditional studies of neuronal avalanches (Beggs and Plenz 2003), which may account for long-range communication in cortex-wide analyses. The cascade size (z) is the total number of active pixels in a given cascade. Next, we examined probability distributions of cascade sizes. A well-studied signature of scale-free dynamics is a cascade size distribution with power-law form. Motivated by previous studies and theory, we used a previously developed (Shew et al. 2009) metric to quantify how closely our measured cascade size distributions resembled power law with exponent −1.5 (Figs. 1*c,d*). A cascade size distribution that is a power law with exponent −1.5 results in κ = 1 (Shew et al. 2009). In contrast, κ > 1 indicates that large cascades are more prevalent, which is expected for certain regimes of dynamics that are not scale-free.

### Scale-Free Activity Patterns Emerge with Wakefulness

**Figure 2.**
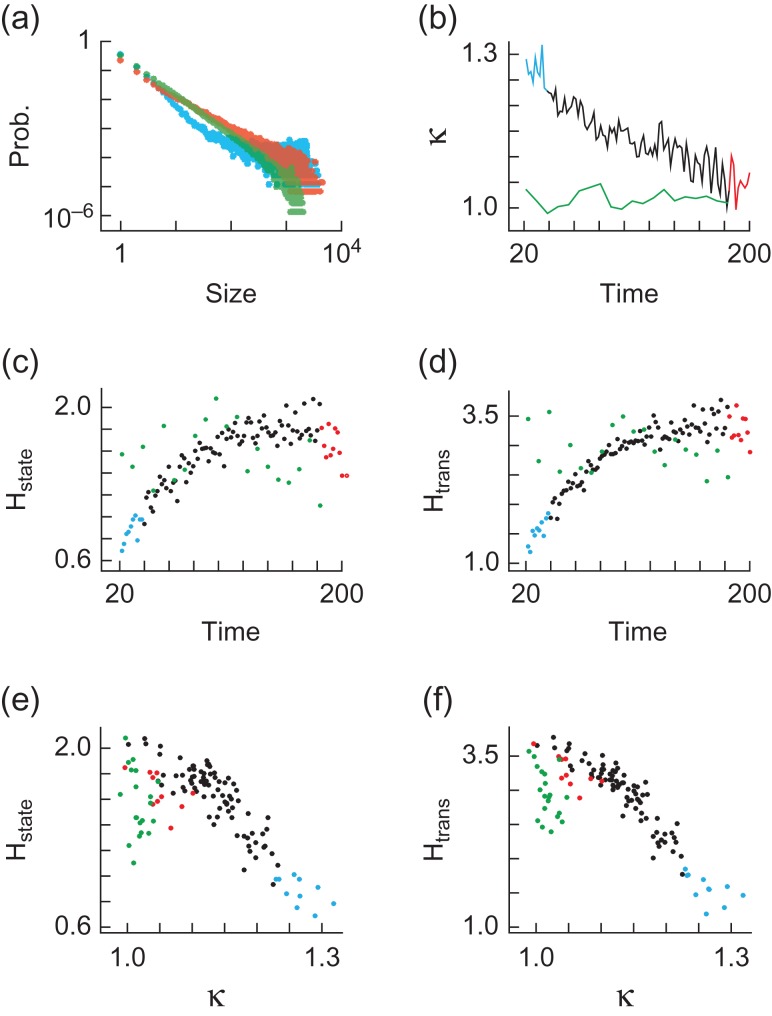
Cortex-wide dynamics with recovery from anesthesia. (*a*) Cascade size (pixels) probability distributions are shown for anesthetized (blue) and awake (red) states from mouse A1 and fully awake results (green) from mouse R1. Blue and red colors correspond to the first and last 20 minutes of data, i.e. anesthetized and awake states. (*b*) κ values as a function of time (minutes) since drug delivery. Color coding and mice as in (a). (*c*) Time (minutes) since drug delivery vs. state visitation entropy H_state_ (bits) derived from the probabilities of visiting each state for k-means clustering algorithm with 10 clusters. Color coding and mice as in (a). (*d*) Time (minutes) since drug delivery vs. state transition entropy H_trans_ (bits) derived from the probabilities of state transitions in a first-order Markov model for k-means clustering algorithm with 10 clusters. Color coding and mice as in (a). (*e*) State visitation entropy H_state_ (bits) as a function of κ. Color coding and mice as in (a). (*f*) State transition entropy H_trans_ (bits) as a function of κ. Color coding and mice as in (a).

We first performed cascade detection across the cortex, without any restriction on the area a cascade is able to cover. We found that the cascade size probability distributions approached power law form P(z)~z^−1.5^ as mice recovered from anesthesia (Fig. 1*a* & Supplementary Fig. 1a), with a change in κ from κ > 1 toward κ ≈ 1, (Fig. 1*b* & Supplementary Fig. 1b), as shown previously (Scott et al. 2014). Cascade detection is always performed for each 50s image sequence independently, with the κ values in Fig. 2*b* calculated from each 50s sequence and the distributions in Fig. 2*a* created from pooled 10 × 50s sequences.

There is an inversely proportional relationship between κ and time since drug delivery (*r* < −0.68, P < 0.001, mice A1, A2, A3). Note that r < −0.68 refers to the fact that the absolute value of r is minimally 0.68 over all animals assessed. This notation is used in the remainder of the study. There is no such relationship for surrogate data (in the following referred to as “noise”) in which each pixel is a random variable with the same power spectrum as the real data and therefore the same rate of active pixels (Supplementary Fig. 2b). Importantly, the absence of relationships in the noise control data indicates that the relationships depend on interactions among pixels and cannot be explained by how fluctuations change at the single-pixel level (Materials and Methods). Moreover, κ was close to 1 and did not change over observation time in fully awake, resting mice (Fig. 2*b* & Supplementary Fig. 1b).

### State Repertoire Increases with Wakefulness

**Figure 3.**
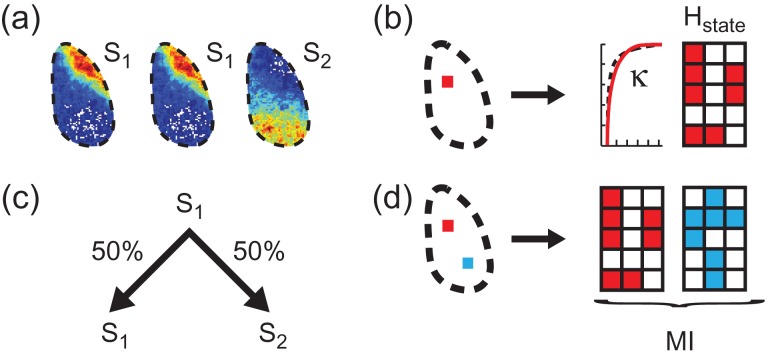
State analysis, κ, entropy and mutual information. (*a*) Three voltage images are assigned state labels S_1_ and S_2_ according to a k-means clustering algorithm. (*b*) Toy model showing a sequence of state indices for an image that consists of 3 pixels evolving over 5 time points. Activity within a single region (red square within the cortex), from which both κ and entropy (H_state_) are calculated. (*c*) Setting up a first-order Markov model for the three-image example in (a), state S_1_ has a 50/50 chance of transitioning to either state S_1_ or S_2_. (*d*) Toy model showing state sequences for a pair of images that each consist of 3 pixels evolving over 5 time points. Activity within two regions (red and blue squares within the cortex) from which their mutual information (MI) is calculated.

We next examined the repertoire (information capacity) of cortical states, defined from patterns of active pixels. To this end, we applied a k-means algorithm to cluster the 2500 activity patterns of each dataset into representative state maps (Fig. 3*a*) (Materials and Methods). We then quantified the state repertoire by calculating the state visitation entropy (H_state_) of the probability distributions P(s_x_) derived from one-dimensional time courses (sequences of state indices) of cortical states, where P(s_x_) is the probability of observing the system in state s_x_, where x = 1,2,…We illustrate an example of such a state sequence in a toy model consisting of 3 pixels evolving over 5 time points (Fig. 3*b*). We found that H_state_ increased as mice recovered from anesthesia (r > 0.65, P < 0.001, mice A1, A2, A3) (Fig. 2*c*), a relationship not observed either for noise (Supplementary Fig. 2c), or for fully awake mice (Fig. 2*c* & Supplementary Fig. 1c). These findings are robust with respect to different numbers of clusters in the k-means algorithm (Supplementary Fig. 1c).

### Diversity of State Transitions Increases with Wakefulness

H_state_ reflects only the number of states visited without taking the order of state visitations into account. This means that H_state_ is identical for the example state sequences A,A,A,B,B,B and A,B,A,B,A,B. Therefore, we also developed a measure of state transition entropy (H_trans_), defined in the context of a first-order Markov model. This means that we considered the conditional probabilities of observing states A,B,… in the next time point, given that we are currently in states A,B,… (Fig. 3*c*). We found that H_trans_ increased as mice recovered from anesthesia (*r* > 0.49, *P* < 0.001, mice A1, A2, A3) (Fig. 2*d* and Supplementary Fig. 1d). There is no such relationship either for noise (Supplementary Fig. 2d), or for fully awake mice (Fig. 2*d* and Supplementary Fig. 1d). These findings are robust with respect to different numbers of clusters in the k-means algorithm (Supplementary Fig. 1d).

### State Repertoire and Diversity of State Transitions are Maximized for Scale-Free Dynamics

We next investigated H_state_ and H_trans_ in the context of cortical dynamics. We found that H_state_ is maximized when cortical dynamics are scale-free, with an inversely proportional relationship between H_state_ and κ for κ > 1 (*r* < −0.67, *P* < 0.01, mice A1, A2, A3, R1, R2) (Fig. 2*e* and Supplementary Fig. 3a). These findings are robust with respect to the number of clusters used (Supplementary Fig. 3a), and are not observed for noise (Supplementary Fig. 2e). Similarly, H_trans_ is maximized at κ ≈ 1, with an inversely proportional relationship between H_trans_ and κ for κ > 1 (*r* < −0.78, *P* < 0.001, mice A1, A2, A3, R1, R2) (Fig. 2*f* and Supplementary Fig. 3b). These findings are robust with respect to the number of clusters in the k-means algorithm (Supplementary Fig. 3b) and are not observed for noise (Supplementary Fig. 2f).

Qualitatively similar results were found when cascades were defined based only on timing of active pixels as in traditional analyses of neuronal avalanches (Beggs and Plenz 2003) (Supplementary Fig. 4).

### Regional State Repertoire Increases with Wakefulness

Above we used k-means clustering of activity over the imaged cortex to show that state repertoire increases with recovery from anesthesia. We proceeded by calculating the information capacity of small regions of cortex, as quantified by their entropy (H), as well as their information transmission, as quantified by the sum of their mutual information (MI) with all other regions.

**Figure 4.**
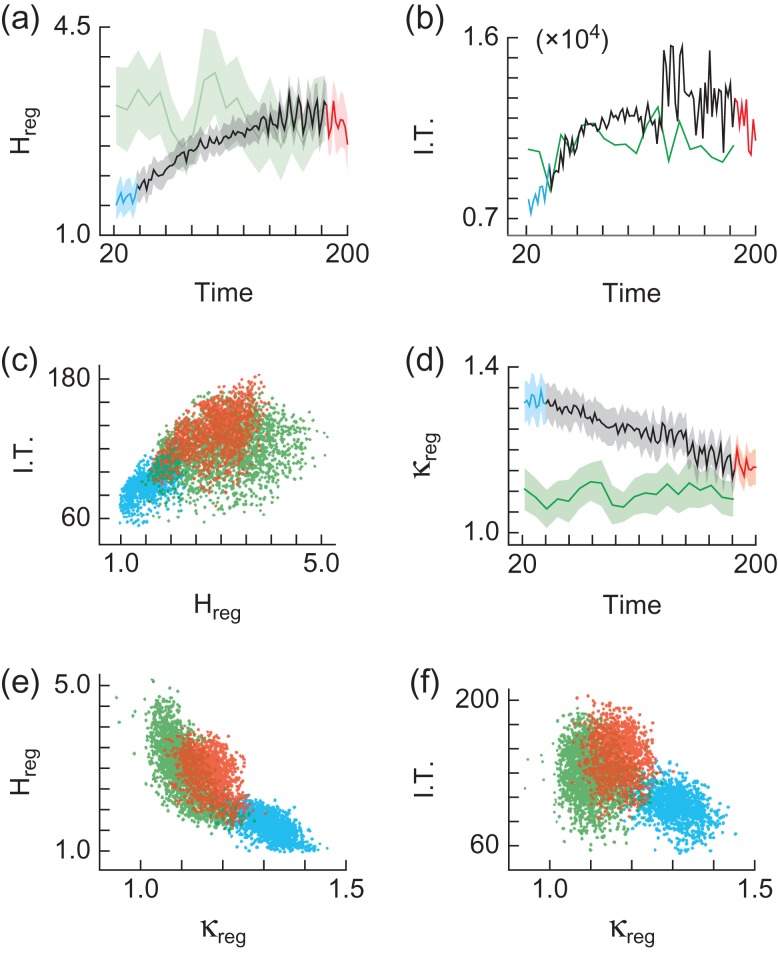
Regional information transmission with recovery from anesthesia. (*a*) Regional entropy H_reg_ (bits) vs. time (minutes) since drug delivery. Shaded region represents standard deviation. Color coding and mice as in Figure 2. (*b*) Time (minutes) since drug delivery vs. information transmission (bits). Color coding and mice as in Figure 2. (*c*) Information transmission (bits), as a function of regional entropy H_reg_ (bits). Color coding and mice as in Figure 2. (*d*) κ_reg_ as a function of time (minutes) since drug delivery. Shaded region represents standard deviation. Color coding and mice as in Figure 2. (*e*) Regional entropy H_reg_ (bits) as a function of κ_reg_. Color coding and mice as in Figure 2. (*f*) Information transmission (bits), as a function of κ_reg_. Color coding and mice as in Figure 2.

We divided the imaged cortex into 8 × 8 pixel regions and calculated the regional information capacity (H_reg_) separately for each region (Fig. 3*b*). We observed a positive relationship between H_reg_ (averaged across all regions) and time since drug delivery (*r* > 0.63, *P* < 0.001, mice A1, A2, A3) (Fig. 4*a*). These results are robust across various combinations of spatiotemporal resolutions (Supplementary Fig. 3c) and no relationship was observed for noise (Supplementary Fig. 5a) or for fully awake mice (Fig. 4*a* & Supplementary Fig. 3c).

The results in Fig. 4 are averaged over several cortical areas (motor, somatosensory, visual, retrosplenial). However, changes in entropy were not uniform over these different areas. For instance, the somatosensory cortex exhibited a larger increase in H than the motor cortex (Supplementary Fig. 6).

### Information Transmission Increases with Wakefulness

We next analyzed the way in which interactions between pairs of cortical regions change as the mice recover from anesthesia. Mutual information (MI) is a measure of the information shared by two regions (*x*,*y*), as described by the reduction in uncertainty about events in region *x* due to knowledge about events in region *y*, shown for a toy model consisting of a pair of state sequences with 3 pixels evolving over 5 time points each (Fig. 3*d*) (Rieke 1997). We corrected for potential subsampling bias and discounted the MI between regions within close proximity to one another (Materials and Methods). We calculate the information transmission (I.T.) for region *x* as the sum of its MI with all other regions IT_*x*_ = Σ_*y*≠*x*_MI(*x*,*y*). We found that I.T. (summed across all regions) increases with time since drug delivery (*r* > 0.49, *P* < 0.001, mice A1, A2, A3) (Fig. 4*b* and Supplementary Fig. 3d). There is no such relationship for noise (Supplementary Fig. 5b) or for fully awake mice (Fig. 4*b* and Supplementary Fig. 3d).

As with entropy, MI also showed some area-dependent changes (Supplementary Fig. 6).

### Information Transmission Increases with Regional State Repertoire

We next investigated the relationship between regional entropy (H_reg_) and information transmission (I.T.) (Fig. 4*c* and Supplementary Fig. 7a). Interpreting the relationship between H_reg_ and I.T. as expressed in Fig. 4c is complicated by the fact that multiple points are re-plotted across different brain states (time since drug delivery) and cortical regions (8 × 8 pixel squares). To resolve this issue we constructed a linear mixed effects model and found that there is a positive relationship between I.T. and H_reg_, while accounting for time and region (t(dof > 390) > 11.24, *P* < 0.001, mice A1, A2, A3, R1, R2), with no such relationship observed for noise.

### Regional State Repertoire and Information Transmission are Maximized for Scale-Free Dynamics

We next investigated how H_reg_ and I.T. are related to how close to scale-free the regional cortical dynamics were, as quantified by κ. We predicted that H_reg_ and I.T. should be maximized for scale-free dynamics (Shew et al. 2011), i.e. when κ ≈ 1. κ_reg_ was calculated from cascade statistics within each 8 × 8 pixel region (Fig. 3b). As with the cortex-wide analysis, κ_reg_ (averaged across all regions) is  > 1 under anesthesia and decreases toward ≈ 1 as mice recover from anesthesia (*r* < −0.96, *P* < 0.001, mice A1, A2, A3) (Fig. 4*d* and Supplementary Fig. 7b). There is no such relationship for noise (Supplementary Fig. 5d) or for fully awake mice (Fig. 4*d* and Supplementary Fig. 7b).

The relationship between κ_reg_ and H_reg_ is complicated by multiple measurements across different brain states and cortical regions. A linear mixed effects model shows an inversely proportional relationship between κ_reg_ and H_reg_, while accounting for time and region (t(dof > 386) < 10.83, *P* < 0.001, mice A1, A2, A3, R1, R2) (Fig. 4*e* and Supplementary Fig. 7c), with no such relationship observed for noise (Supplementary Fig. 5e).

Finally, a linear mixed effects model shows an inversely proportional relationship between κ_reg_ and I.T., while accounting for time and region (Fig. 4*f* and Supplementary Fig. 7d) (t(dof > 386) < −3.43, *P* < 0.001, mice A1, A2, A3, R2), with no such relationship observed for noise (Supplementary Fig. 5f).

## Discussion

In summary, we measured information capacity and information transmission across the cortex in mice recovering from anesthesia, using GEVI optical imaging with  < 100 μm spatial resolution. We found that both information capacity and information transmission increased steadily as the mice awoke, reaching their highest levels in the awake state, in line with previous findings in primates (Fekete et al. 2009). We also found that levels of information capacity and information transmission were not uniformly distributed across the cortex. Some regions had higher information capacity and information transmission, while others were lower. We next sought aspects of cortical dynamics that could explain this regional variability and found that information capacity and information transmission were highest for regions with scale-free dynamics (κ~1). The “scale-free-ness” of the dynamics (i.e. κ) accounted both for the cortex-wide rise over time, as well as for the region-to-region variability in these measures of information processing, during recovery from anesthesia. Our results constitute the first *in vivo* confirmation of predictions that information capacity and information transmission are maximized for scale-free dynamics.

Our results here and our other recent work (Scott et al. 2014) support the hypothesis that the awake cortex operates near a dynamical regime called “criticality”. The criticality hypothesis states that the awake brain operates close to a second-order phase transition, poised at the boundary between ordered and chaotic neural dynamics (Plenz and Thiagarajan 2007; Chialvo 2010; Beggs and Timme 2012). Previous studies suggest that maintaining a network of neurons at criticality requires a balance of excitatory and inhibitory synaptic interactions (Shew et al. 2011; Wang et al. 2011; Gautam et al. 2015) and can depend on network structure (Larremore et al. 2011; Wang and Zhou 2012). One of the implications of this hypothesis is that, at criticality, network dynamics should be scale-free. Thus, one may interpret our measure κ as indicating proximity to criticality. However, caution is called for with such interpretations; a scale-free cascade size distribution is a necessary, but insufficient condition to prove the system is at criticality. Indeed, scale-free statistics can arise from mechanisms other than criticality (Beggs and Timme 2012; Stumpf and Porter 2012). Nonetheless, a second implication of the criticality hypothesis is that, at criticality, information transmission should be optimized across multiple scales—from individual neurons, to local circuits and entire cortical regions. Our observation of high information transmission near κ = 1 is in line with this possibility.

Previous investigations of the criticality hypothesis have utilized techniques that either have wide cortical coverage and low spatial resolution, such as electroencephalography (EEG) (Meisel et al. 2013) and functional magnetic resonance imaging (fMRI) (Tagliazucchi et al. 2012), or low cortical coverage with high spatial resolution, such as multi-electrode arrays (Shew et al. 2009) and calcium imaging (Bellay et al. 2015). However, to gain insight into information transmission among multi-scale cortical circuits, one must first be able to perform measurements with both wide coverage and high spatial resolution. We met this criterion using genetically encoded voltage indicator (GEVI) optical imaging. In this context, our findings are the first supporting evidence for the hypothesis that information transmission among cortical regions is maximized near criticality.

Other recent *in vivo* studies of the transition from anesthetized to awake states found an emergence of scale-free activity (Bellay et al. 2015; Solovey et al. 2015). These studies showed that this transition is accompanied by increasing irregularity in firing patterns, consistent with higher information capacity. However, unlike these previous studies, the high coverage and spatiotemporal resolution of our voltage imaging data enabled us to simultaneously probe the states of different cortical regions. We found that different regions of cortex do not operate in the same dynamical regime, i.e. with different κ values, which we interpret as different degrees of proximity to criticality. As we showed in previous work (Scott et al. 2014), the motor cortex exhibits systematically higher κ values compared with the somatosensory cortex, although both areas shift from κ > 1 toward κ = 1 during recovery from anesthesia. Here we found that the motor cortex had systematically lower entropy compared to the somatosensory cortex, in line with the idea that the highest entropy is found near κ = 1 (Supplementary Fig. 6). This is consistent with another recent study (Shew et al. 2015), which showed that sensory input can cause changes in cortical state in a region specific manner. Such regional variability may explain why other studies that tested single small areas of human, monkey and cat cortex did not observe scale-free spike activity (Dehghani et al. 2012). Nonetheless, we found that all cortical regions shift toward scale-free activity during recovery from anesthesia, in line with a previous study showing signatures of criticality emerging in the cortical dynamics of rats recovering from anesthesia (Ribeiro et al. 2010). The cortical dynamical regime can also vary significantly depending on arousal, attention, body motility and other behavioral parameters (Fontanini and Katz 2008; Harris and Thiele 2011; Renart and Machens 2014). An open challenge for future work is to determine how such behaviorally driven state changes are related to information capacity and information transmission.

We emphasize that our metrics of information capacity (entropy) and transmission (mutual information) are meaningful only as relative measures. The absolute values depend on a range of factors, including the volume of neurons subtended by each pixel and the area of the imaged cortex. As such, we are not deriving an absolute measure of the information that a cortical region can represent or transmit and it is therefore not appropriate to directly compare values among mice, or to values reported in other studies (Shew et al. 2011).

Our results complement previous work that relates the action of general anesthetics to measures of information processing (Tononi et al. 1998; Alkire et al. 2008). For example, several general anesthetics produce deep unconsciousness with stereotypic, global on–off patterns of neural activity that traverse the cortex. This effect appears to cause a loss of distinguishable firing patterns (loss of information capacity), limiting the brain's ability to effectively integrate information (Alkire et al. 2008). Indeed, integrated information theory argues that the maintenance of consciousness is directly dependent on the integration of information in the brain (Tononi and Koch 2015). Our results support this view, in that our mice recovered from anesthesia while showing increased levels of information capacity and information transmission. Moreover, our results demonstrate that raising the information capacity and transmission among different cortical regions is associated with mechanisms that tune the regions toward scale-free dynamics.

## Supplementary Material

Supplementary material can be found at: http://www.cercor.oxfordjournals.org/.

## Funding

This work was supported by the Medical Research Council (E.D.F.), the Wellcome Trust and GlaxoSmithKline (G.S.), the Foundational Questions Institute (W.L.S.), the Human Frontier Science Program (C.S. and T.K.), RIKEN, Japan (T.K.) and National Institute for Health Research Professorship - RP-011-048 (D.J.S.).

## Supplementary Material

Supplementary Data
