# Supplementary material for: Cortical Entropy, Mutual Information and Scale-Free Dynamics in Waking Mice
Source: Cereb Cortex. 2016 Sep 19;26(10):3945–52. doi: 10.1093/cercor/bhw200 (PMC5028006; doi:10.1093/cercor/bhw200)
Supplement: Supplementary Data [file Supp_Figs.pdf]

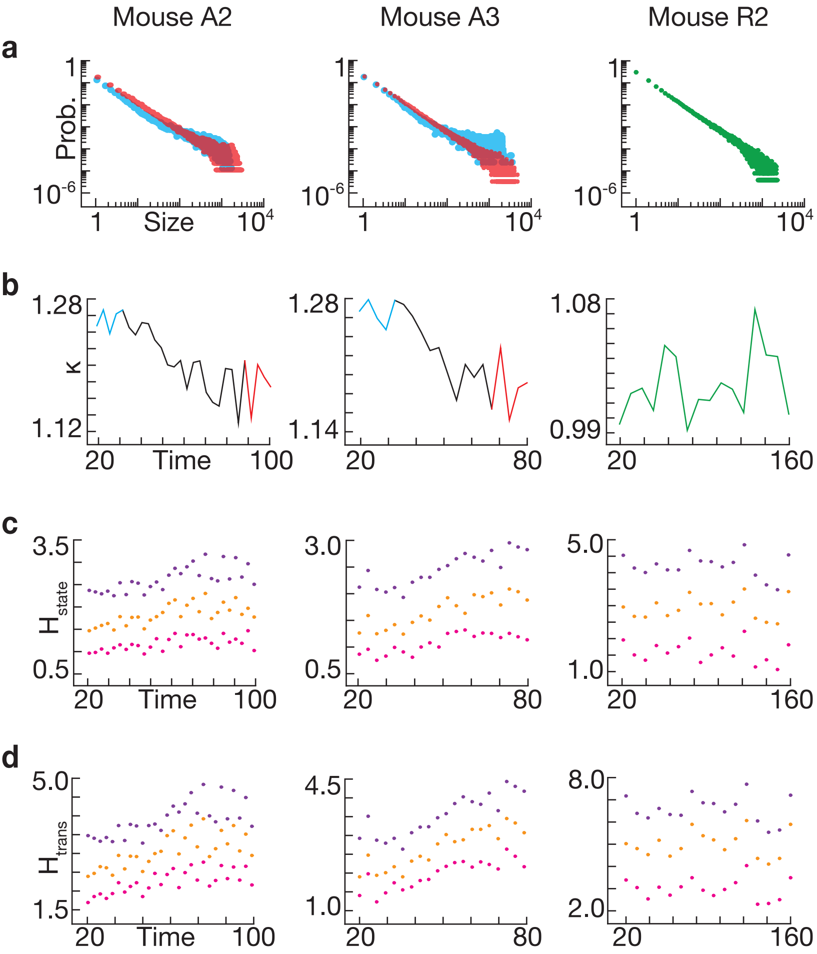

Supplementary Figure 1: **(a)** Size (pixels) probability distributions. Two mice recovering from anesthesia (A2 and A3) and one fully awake mouse (R2) shown from left to right in all panels. Colour coding as in Figure 2. **(b)** κ values as a function of time (minutes). Colour coding and mice as in **(a)**. **(c)** State entropy H_{state} (bits) as a function of time (minutes). Mice as in **(a)**. Pink, orange and purple colours correspond to 10, 50 and 200 clusters in the k-means algorithm. **(d)** Transition entropy H_{trans} (bits) from a 1st order Markov model as a function of time (minutes). Colour coding and mice as in **(c)**.

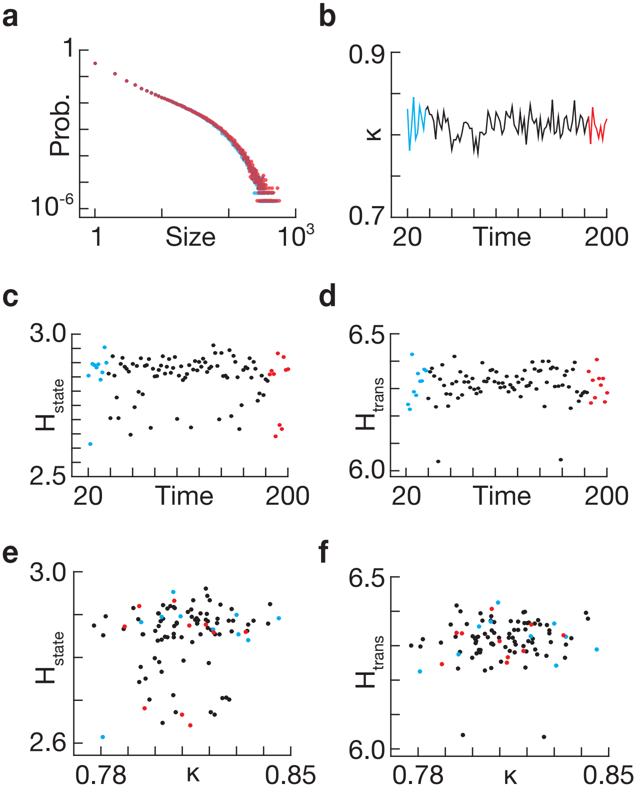

Supplementary Figure 2: Results for noise data corresponding to layout and colour coding in **Figure 2** for mouse A1.

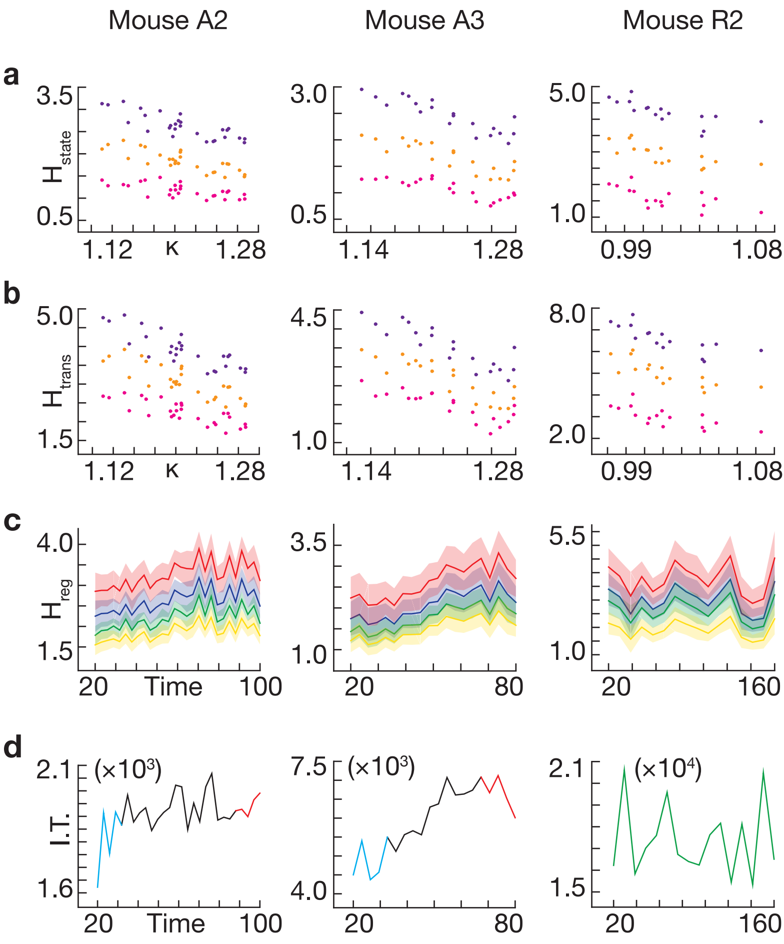

Supplementary Figure 3: **(a)** State entropy H_{state} (bits) as a function of κ. Colour coding and mice as in **Supp. Fig. 1 (c)**. **(b)** Transition entropy H_{trans} (bits) from a 1st order Markov model as a function of κ. Colour coding and mice as in **Supp. Fig. 1 (c)**. **(c)** Regional entropy H_{reg} (bits) as a function of time (minutes). Mice as in **Supp. Fig. 1**. H_{reg} is calculated for four different cortex grid definitions. Red: each square consists of 8×8 pixels for half temporal resolution data. Blue: each square consists of 8×8 pixels. Green: each square consists of 5×5 pixels for half spatial resolution data. Yellow: each square consists of 5×5 pixels. **(d)** Information transmission (bits), as a function of time (minutes). Colour coding and mice as in **Supp. Fig. 1 (a)**.

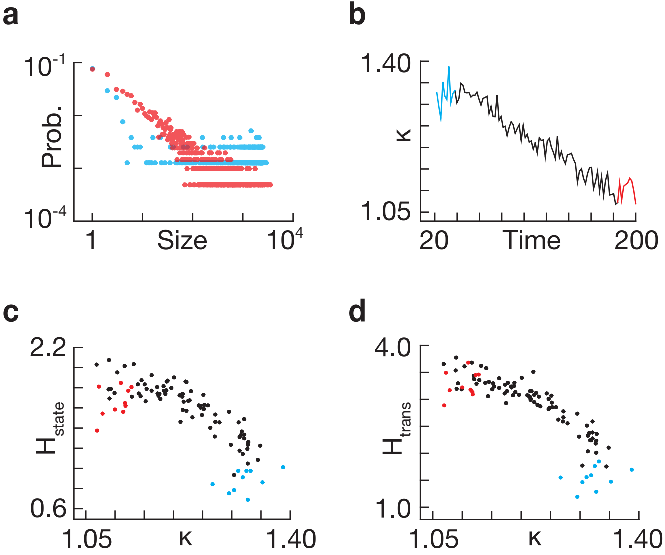

Supplementary Figure 4: Results for purely temporal cascade detection corresponding to layout and colour coding in **Figure 2 (a)**, **(b)**, **(e)** & **(f)** for mouse A1.

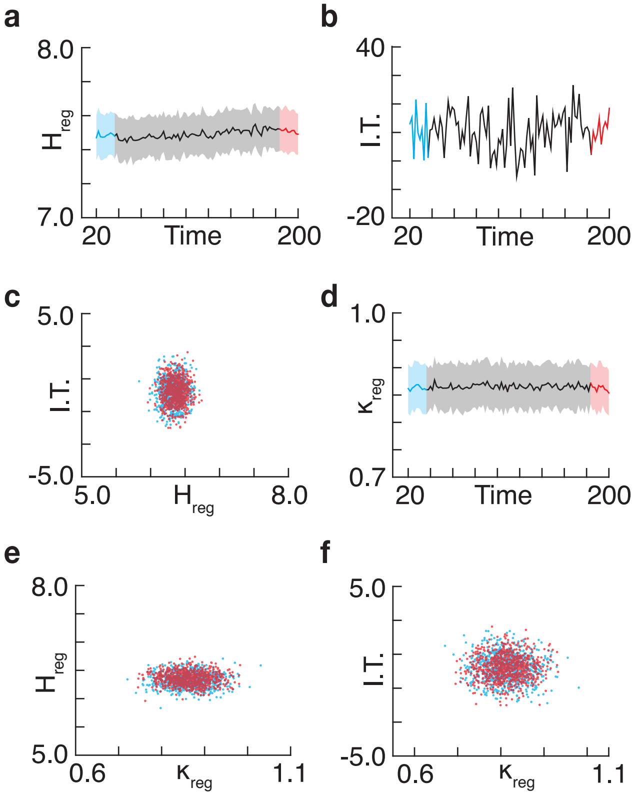

Supplementary Figure 5: Results for noise data corresponding to layout and colour coding in **Figure 4** for mouse A1.

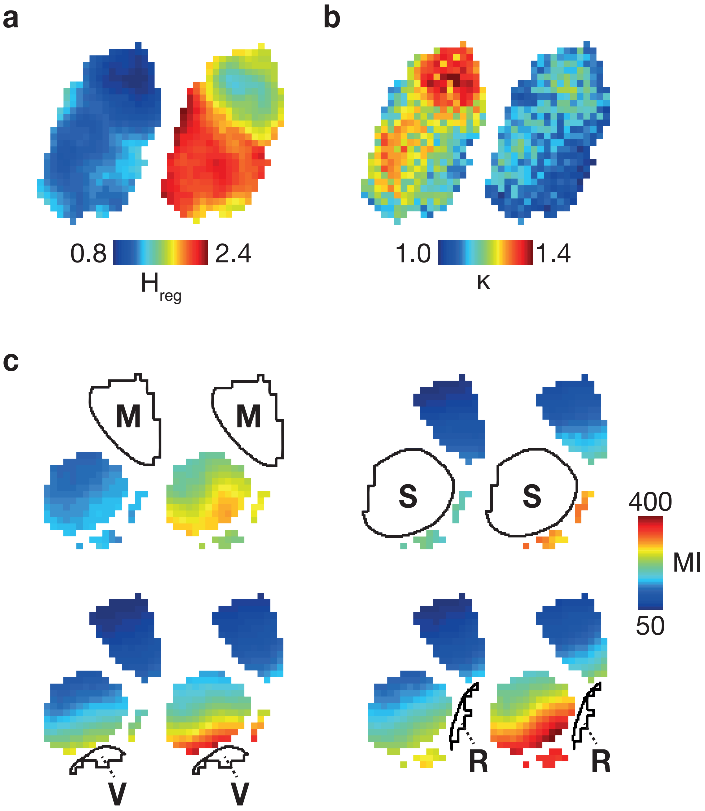

Supplementary Figure 6: **(a)** H_{reg} (bits) within 5×5 pixel regions for anaesthetised (left) and awake (right) states. **(b)** κ_{reg} within 5×5 pixel regions for anaesthetised (left) and awake (right) states. **(c)** Mean values of mutual information between all regions in the motor (top left), somatosensory (top right), visual (bottom left) and retrosplenial (bottom right) cortices and the rest of the brain for anaesthetized (left) and awake (right) states.

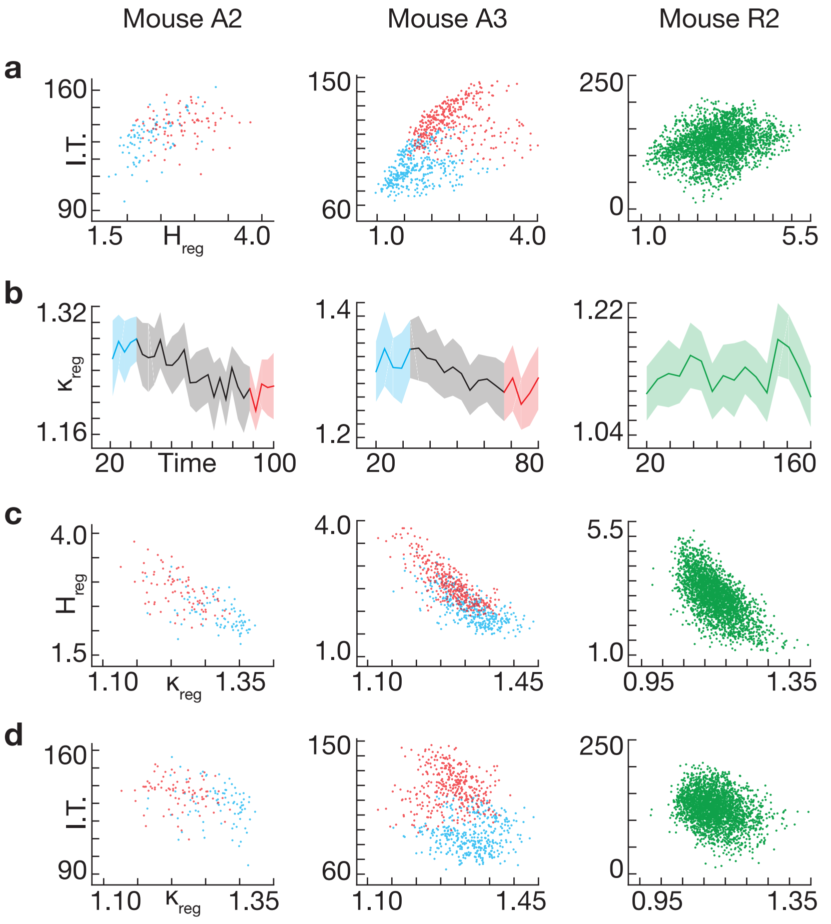

Supplementary Figure 7: **(a)** Information transmission (bits), as a function of regional entropy H_{reg} (bits). Colour coding and mice as in **Supp. Fig. 1 (a)**. **(b)** κ_{reg} as a function of time (minutes). Colour coding and mice as **Supp. Fig. 1 (a)**. **(c)** Regional entropy H_{reg} (bits) as a function of κ_{reg}. Colour coding and mice as in **Supp. Fig. 1 (a)**. **(d)** Information transmission (bits), as a function of κ_{reg}. Colour coding and mice as in **Supp. Fig. 1 (a)**.
